# Nucleosomes of polyploid trophoblast giant cells mostly consist of histone variants and form a loose chromatin structure

**DOI:** 10.1038/s41598-018-23832-2

**Published:** 2018-04-11

**Authors:** Koji Hayakawa, Kanae Terada, Tomohiro Takahashi, Hidehiro Oana, Masao Washizu, Satoshi Tanaka

**Affiliations:** 1 Laboratory of Cellular Biochemistry, Department of Animal Resource Sciences/Veterinary Medical Sciences, The University of Tokyo, Tokyo, Japan; 2 Department of Mechanical Engineering, The University of Tokyo, Tokyo, Japan; 3 Department of Bioengineering, The University of Tokyo, Tokyo, Japan

## Abstract

Trophoblast giant cells (TGCs) are one of the cell types that form the placenta and play multiple essential roles in maintaining pregnancy in rodents. TGCs have large, polyploid nuclei resulting from endoreduplication. While previous studies have shown distinct gene expression profiles of TGCs, their chromatin structure remains largely unknown. An appropriate combination of canonical and non-canonical histones, also known as histone variants, allows each cell to exert its cell type-specific functions. Here, we aimed to reveal the dynamics of histone usage and chromatin structure during the differentiation of trophoblast stem cells (TSCs) into TGCs. Although the expression of most genes encoding canonical histones was downregulated, the expression of a few genes encoding histone variants such as H2AX, H2AZ, and H3.3 was maintained at a relatively high level in TGCs. Both the micrococcal nuclease digestion assay and nucleosome stability assay using a microfluidic device indicated that chromatin became increasingly loose as TSCs differentiated. Combinatorial experiments involving H3.3-knockdown and -overexpression demonstrated that variant H3.3 resulted in the formation of loose nucleosomes in TGCs. In conclusion, our study revealed that TGCs possessed loose nucleosomes owing to alterations in their histone composition during differentiation.

## Introduction

The rodent placenta consists of a labyrinth zone and junctional zone. The latter is further divided into the spongiotrophoblast and trophoblast giant cell (TGC) layers^1–3^. TGCs secrete various proteins such as extracellular matrix components, cell adhesion molecules, cytokines, and hormones to promote embryo implantation and maternal adaptations during pregnancy^2^. TGCs are characterized by their large cytoplasm and polyploid nuclei resulting from endoreduplication. In contrast to proliferating diploid cells, TGCs undergo repeated DNA replication without mitosis and accumulate more than 1,000 copies of genomic DNA^4,5^. Recently, it was revealed that there were a few under- and over-replicated regions in the TGC genome, which suggested that their genomic DNA formed a polytene structure^6,7^. To organize such an unusual genome, TGCs are expected to possess a unique chromatin structure that has not been explored in details.

The structural organization of eukaryotic DNA in chromatin is intricately linked to the regulation of many essential cellular processes, such as genomic DNA replication and transcription^8,9^. Nucleosomes are the structural units of chromatin and are dynamically remodeled, assembled, and disassembled by histone chaperones^10,11^. Genomic DNA is packaged by the core histones, namely H2A, H2B, H3, and H4, and length of the linker DNA bound to the linker histone H1 varies^12,13^.

Histones are classified into two types, canonical histones and histone variants (non-canonical histones), based on the similarity of amino acid sequences and their cell cycle-dependent or independent expression^14–16^. Genes for canonical histones form clusters on certain chromosomes and code for few variations in the amino acid sequences^15,17^. In contrast, genes for histone variants are located away from the gene clusters and code for higher variations in amino acid sequences than canonical histones^15,16^. The most pronounced sequence divergence is found in H2A, leading to the highest number of variants. Both H2B and H3 have a few variations in their sequences, although there is no identified variant for H2B. In contrast, H4 neither displays sequence divergence nor has any identified variants^14,15^. High-order chromatin organization in mammals is modulated by several epigenetic mechanisms, including DNA methylation and histone post-translational modifications^18–20^. Since there is a wide variety of histones, chromatin structure might change by changing the combination of histones.

In the present study, we aimed to reveal the dynamics of chromatin structure and histone usage during TGC differentiation. For this, we examined the nucleosomal content, chromatin structure, and histone mobility of TGCs using a trophoblast stem cell (TSC) differentiation system^21^, and observed that TGCs had loose nucleosome structures incorporated with histone variants, such as H2AX and H3.3.

## Results

### The repertoire of histones in TGCs is limited compared to that in undifferentiated cells

**Figure 1:**
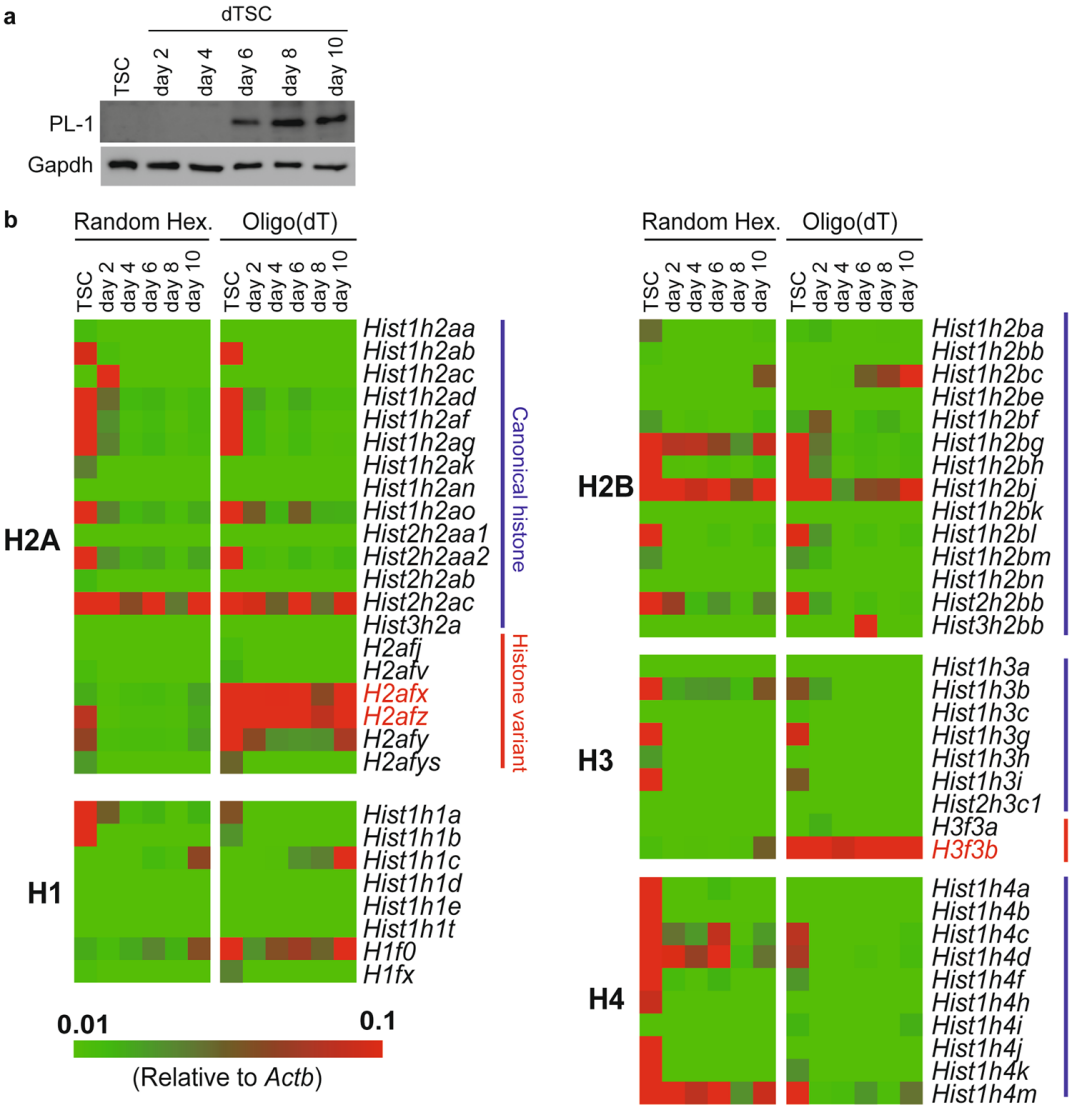
The variation in canonical histone subtypes and histone variants decreases during differentiation of trophoblast stem cells (TSCs). (**a**) Western blot analysis showing the protein level of trophoblast giant cell (TGC) marker PL-I in TSCs and differentiated TSCs (dTSCs). Cytoplasmic protein was used. Gapdh was used as an internal control. (**b**) The mRNA levels of histone-coding genes were evaluated using RT-qPCR, normalized to *Actb* expression, and visualized as a heatmap. Both random hexamers and oligo(dT) were used as primers for cDNA synthesis. Color scale bars indicate the expression level of each gene relative to that of *Actb*. Genes encoding histone variants described in Fig. 2 are shown in red.

TSCs were induced to differentiate and collected every other day till day 10 of differentiation. Expression of PL-I protein, a specific marker for TGCs, was detected after day 6 of differentiation using western blotting (Fig. 1a), indicating that differentiated TSCs (dTSCs) could be regarded as TGCs as early as day 6 of differentiation in terms of hormone expression.

To reveal the histone composition of TGC nucleus, the expression of histone-coding genes (20, 14, 9, 10, and 8 genes for H2A, H2B, H3, H4, and H1, respectively) was investigated using RT-quantitative PCR (RT-qPCR). Since mRNAs of some histone-coding genes have a stem–loop structure but no poly-A tail at their 3′ UTR^14,22,23^, we used random hexamers and oligo(dT) as primers for cDNA synthesis. The qPCR analysis revealed that mRNAs of ten H2A genes (*Hist1h2ab*, *Hist1h2ad*, *Hist1h2af*, *Hist1h2ag*, *Hist1h2ao*, *Hist2h2aa2*, *Hist2h2ac*, *H2afx*, *H2afz*, and *H2afy*), five H2B genes (*Hist1h2bg*, *Hist1h2bh*, *Hist1h2bj*, *Hist1h2bl*, and *Hist2h2bb*), four H3 genes (*Hist1h3b*, *Hist1h3g*, *Hist1h3i*, and *H3f3b*), nine H4 genes (*Hist1h4a–d*, *Hist1h4f*, *Hist1h4h*, *Hist1h4j*, *Hist1h4k*, and *Hist1h4m*), and three H1 genes (*Hist1h1a*, *Hist1h1b*, and *H1f0*) were highly expressed in the undifferentiated state (Fig. 1b). Interestingly, most genes showing high expression in TSCs were downregulated during differentiation. In contrast, expression of three out of ten H2A genes (*Hist2h2ac*, *H2afx*, and *H2afz*), two out of five H2B genes (*Hist1h2bg* and *Hist1h2bj*), one out of four H3 genes (*H3f3b*), three out of nine H4 genes (*Hist1h4c*, *Hist1h4d*, and *Hist1h4m*), and one out of three H1 genes (*H1f0*) remained relatively high throughout differentiation. Expression of *Hist1h2bc* and *Hist1h1c* was observed at day 6 of differentiation in dTSCs. Thus, the complexity of histones in dTSC nuclei was markedly lower compared to that in TSCs.

### Histone variants H2AX and H3.3 predominantly occupy the chromatin of TGCs

**Figure 2:**
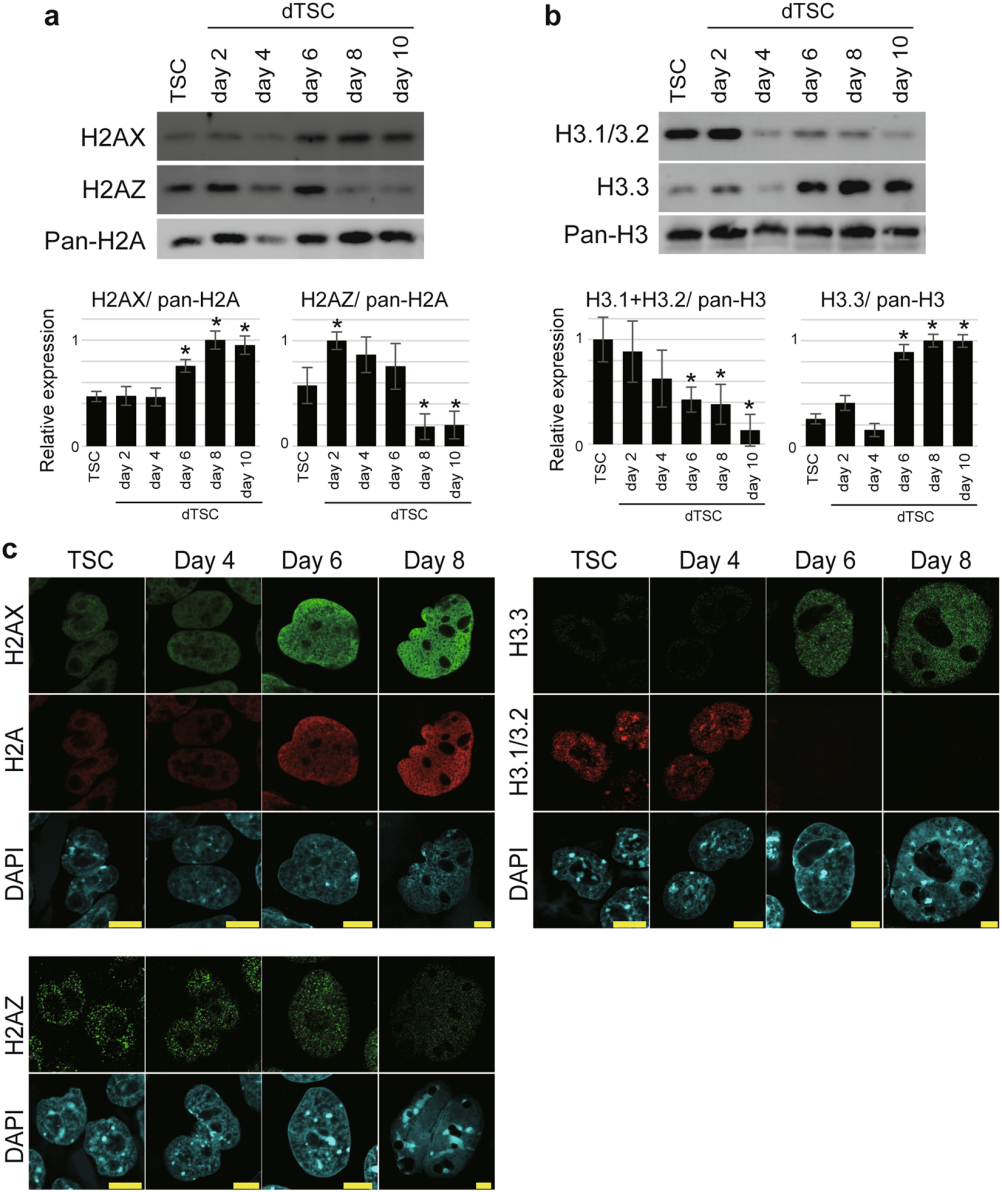
The histone variants H2AX, H2AZ, and H3.3 are predominantly present in TGC chromatin. (**a,b**) Protein levels of the H2A variants H2AX, H2AZ (**a**), and canonical H3 H3.1/3.2, and the H3 variant H3.3 (**b**) in TSCs and dTSCs. Upper panel shows western blotting results for H2AX, H2AZ, pan-H2A (**a**), H3.1/3.2, H3.3 and pan-H3 (**b**). Western blotting was performed using total histones equivalent to 0.5 μg of genomic DNA. Bottom panel shows H2AX, H2AZ, H3.1/3.2, and H3.3 levels in dTSCs, which were normalized to the pan-H2A (**a**) or pan-H3 level (**b**) (Fig. S1). The values were expressed as a ratio relative to the maximum average. The mean ± standard deviation (S.D.) of three independent experiments are shown. *P < 0.05 versus TSC (Student’s *t*-test). (**c**) Immunofluorescence staining of H2AX, H2AZ, H3.1/3.2, and H3.3 in TSCs and dTSCs at day 4, 6, and 8 of differentiation. Bars = 10 μm.

Since the expression of *H2afx* (encoding H2AX), *H2afz* (encoding H2AZ), and *H3f3b* (encoding H3.3) was maintained at a high level throughout the differentiation of TSCs (Fig. 1b), the protein levels of H2AX, H2AZ, H3.3, and the canonical histone H3.1/3.2 were estimated using western blot analysis by normalizing the intensity values of each band to those of pan-H2A or -H3 levels (Fig. S1). The level of H2AX increased from day 6 and plateaued at day 8 (Fig. 2a). For H2AZ, expression increased by day 2 and remained high until day 6; however, it drastically decreased to less than half of the level observed for TSC after day 8 (Fig. 2a). Notably, the level of H3.3 increased at day 6, in contrast to that of the canonical type H3.1/3.2 (Fig. 2b).

To reveal their subnuclear localization, immunofluorescence staining was performed. H2AX and H2AZ were detected broadly in the nucleoplasm but not in the nucleolus, and H2AZ signals were observed as dotted foci compared to H2AX signals. The intensity of H2AX increased as the nucleus expanded throughout differentiation (Fig. 2c). The intensity of the H2AZ signal remained constant until day 6 of differentiation and reduced remarkably at day 8 (Fig. 2c). In contrast to TSCs and dTSCs at day 4, H3.3 could be detected in dTSC nuclei at day 6 and 8, while H3.1/3.2 was barely detectable (Fig. 2c). The dTSCs with a small nucleus (non-TGCs, most likely spongiotrophoblast or syncytiotrophoblast cells) at days 6 and 8 of differentiation exhibited a lower expression of H2AX and H3.3 than putative TGCs (Fig. S2).

**Figure 3:**
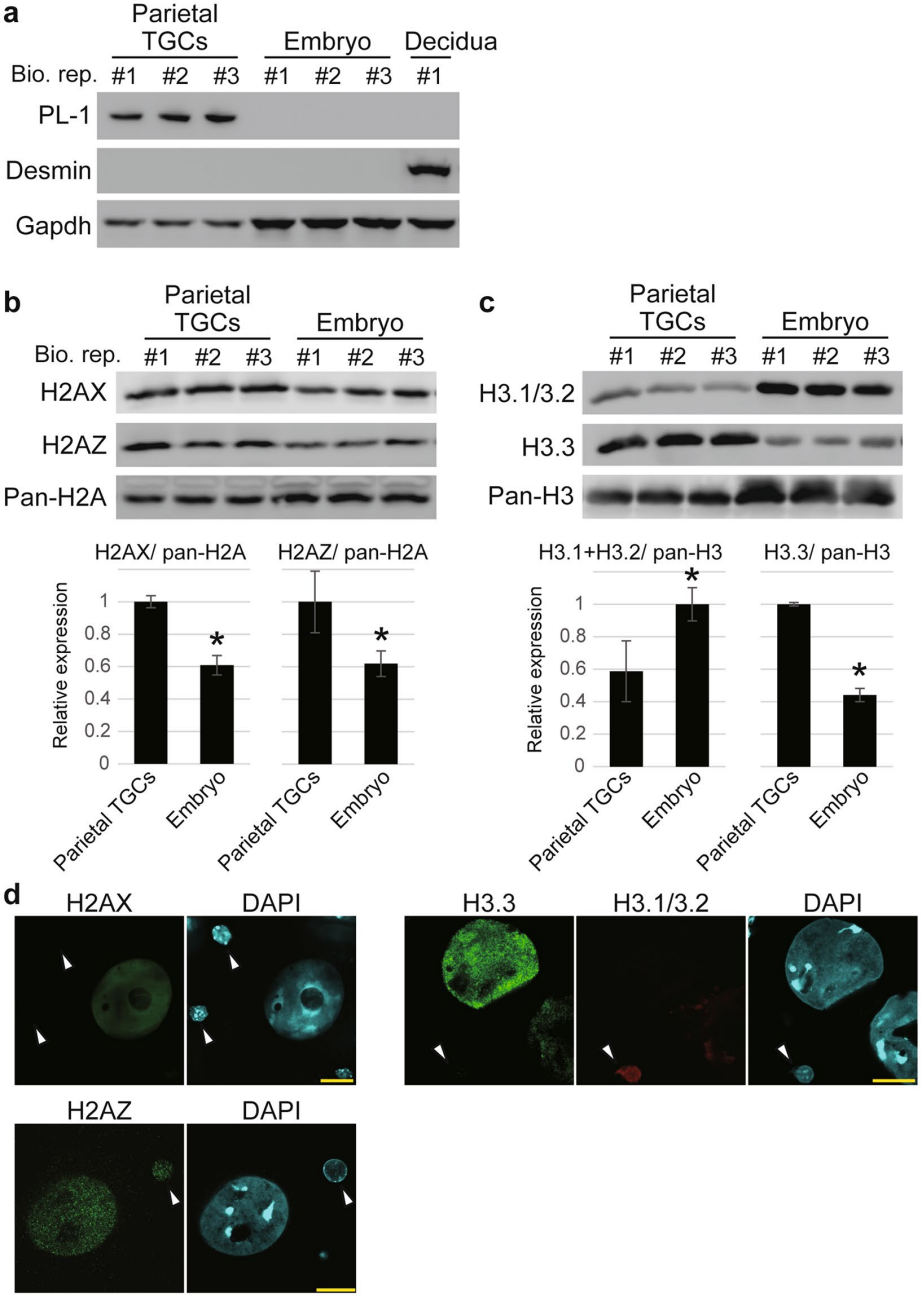
Expression status of histone variants H2AX, H2AZ and H3.3 in TGCs *in vivo*. (**a**) Western blotting analysis of TGC marker PL-1 and decidual marker Desmin in the cytoplasmic protein samples. Bio. Rep. indicates biological replicates. (**b**,**c**) Western blotting analysis of H2AX, H2AZ (**b**), H3.1/3.2, and H3.3 (**c**) in the nuclear protein of parietal TGCs and embryos. The graphs below the blots show H2AX, H2AZ, H3.1/3.2, and H3.3 levels in parietal TGCs and embryos, which were normalized to the pan-H2A or pan-H3 level. The values were expressed as a ratio relative to the maximum average. The mean ± S.D. of three independent experiments are shown. *P < 0.05 versus parietal TGCs (Student’s *t*-test). (**d**) Immunofluorescence staining of H2AX, H2AZ, H3.1/3.2, and H3.3. White arrowheads indicate diploid cells in Reichert’s membrane. Bars = 20 μm.

To investigate the expression of aforementioned histone variants in TGCs *in vivo*, we collected parietal TGCs and embryos at the embryonic day 9.5 (E9.5) (Fig. 3a). Western blotting analysis revealed that the expression of H2AX, H2AZ, and H3.3 was significantly higher in parietal TGCs than that in embryos, in contrast to that of canonical H3.1/3.2 (Fig. 3b and c). H2AX and H3.3 signals were distributed in the entire nucleoplasm in parietal TGCs. These two histone variants were undetectable in diploid cells, most probably in parietal endodermal cells present in the Reichert’s membrane (Fig. 3d). Similar H2AZ signal intensities were detected in parietal TGCs, as well as diploid cells. The expression status of these histone variants indicates that dTSCs at day 6 of differentiation recapitulate the features of TGCs *in vivo*.

Taken together, our results showed that during the early stage, dTSCs predominantly expressed H2AZ and H3.1/H3.2; however, dTSCs after day 6 of differentiation and parietal TGCs *in vivo* predominantly expressed the H2AX and H3.3 isoforms/variants of H2A and H3, respectively. Since these histones are categorized as histone variants, which have unique functions in the formation of loose nucleosome^16^, we hypothesized that differentiation towards TGCs potentially destabilizes the histone–histone and histone–DNA interactions in nucleosomes.

### TGCs have loose nucleosome structures

**Figure 4:**
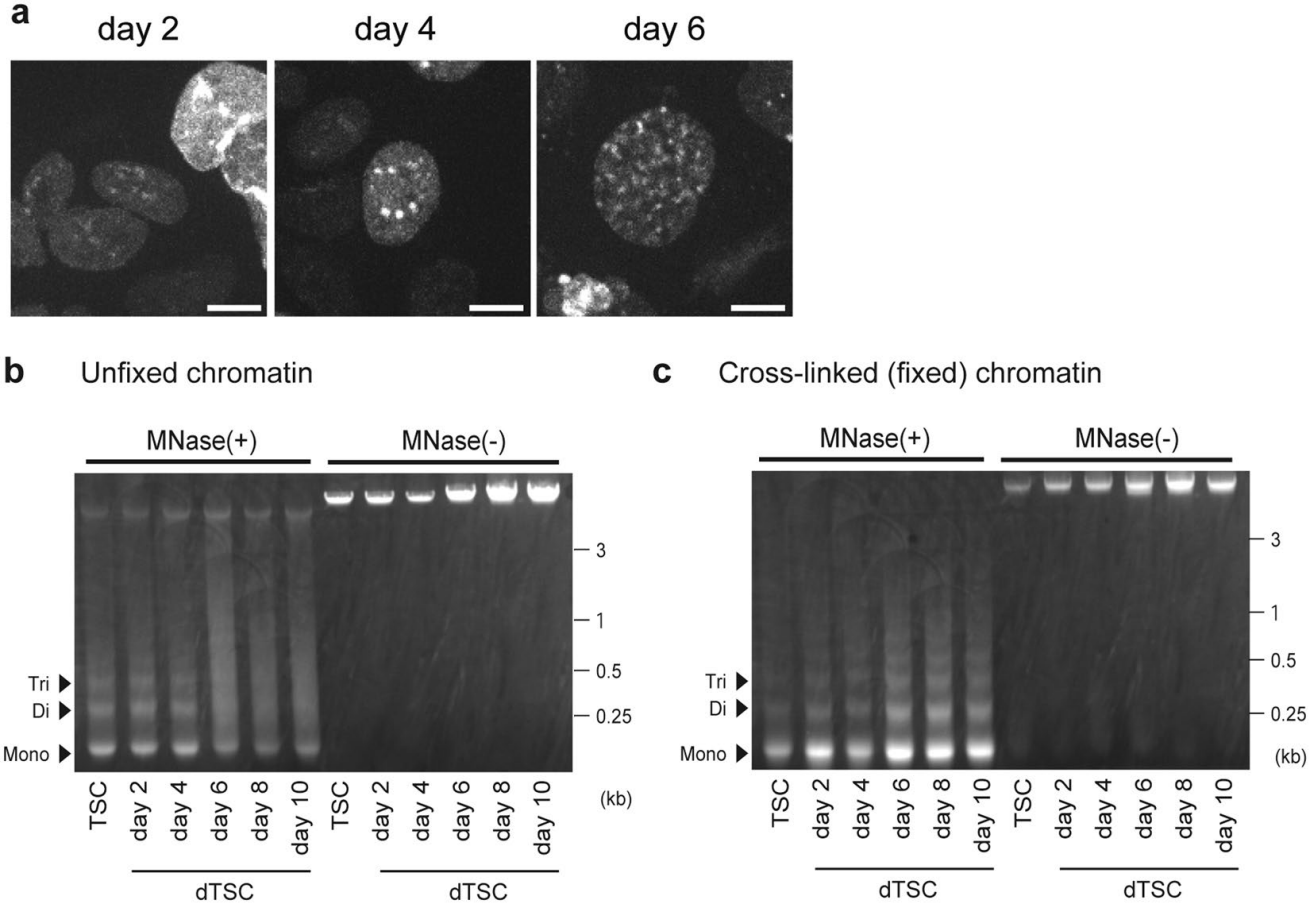
Nuclei of TGCs containing loose chromatin. (**a**) Live cell imaging for H4-GFP during differentiation. Representative snapshots from a live cell movie (Movie S1), which was recorded for 96 h (tracking the same field), are shown. Bars = 5 μm. (**b**,**c**) MNase digestion analysis using chromatin from TSCs and dTSCs. Unfixed (**b**) or fixed (**c**) chromatin was digested with MNase and electrophoresed on an agarose gel with (+) MNase and without (−) MNase as the negative control. Mono, mono-nucleosome; Di, di-nucleosomes; Tri, tri-nucleosomes.

To visualize chromatin dynamics in a single cell, a mouse TSC line stably expressing GFP-fused H4 (H4-GFP) was established and GFP fluorescence was monitored for 96 h during TSC differentiation (days 2–6 of differentiation). A majority of the cells underwent cell division three times within the first 48 h (days 2–4 of differentiation), followed by the expansion of nuclear size (Fig. 4a and Movie S1) most likely due to endoreduplication. After the last cell division, GFP foci, possibly representing heterochromatin, became apparent and gradually increased in number with the simultaneous appearance of a GFP-poor area (i.e., areas with less nucleosomes).

The micrococcal nuclease (MNase) digestion assay using unfixed chromatin demonstrated that TSCs and dTSCs at days 2 and 4 of differentiation showed typical nucleosomal structures, since bands corresponding to mono-, di-, and tri-nucleosomal sizes were observed (Fig. 4b). A band of barely digested DNA (>3 kb) also appeared in both TSCs and dTSCs. Remarkably, after day 6 of differentiation, digested genomic DNA did not show obvious bands for di- and tri-nucleosomes, but showed blurred bands instead. The MNase digestion assay using fixed chromatin confirmed that after day 6 of differentiation, like TSCs, dTSCs also formed nucleosomal structures (Fig. 4c). Of note, di- and tri-nucleosomal bands were lower in dTSCs after day 6 than in TSCs and dTSCs at days 2–4 (Fig. S3), which may reflect the difference of structures of linker DNA regions. These results using unfixed and fixed chromatin indicated that not only the linker DNA regions, which flank the nucleosome core, but also the DNA surrounding histone core was more easily digested by MNase after day 6 of differentiation in dTSCs than in TSCs.

**Figure 5:**
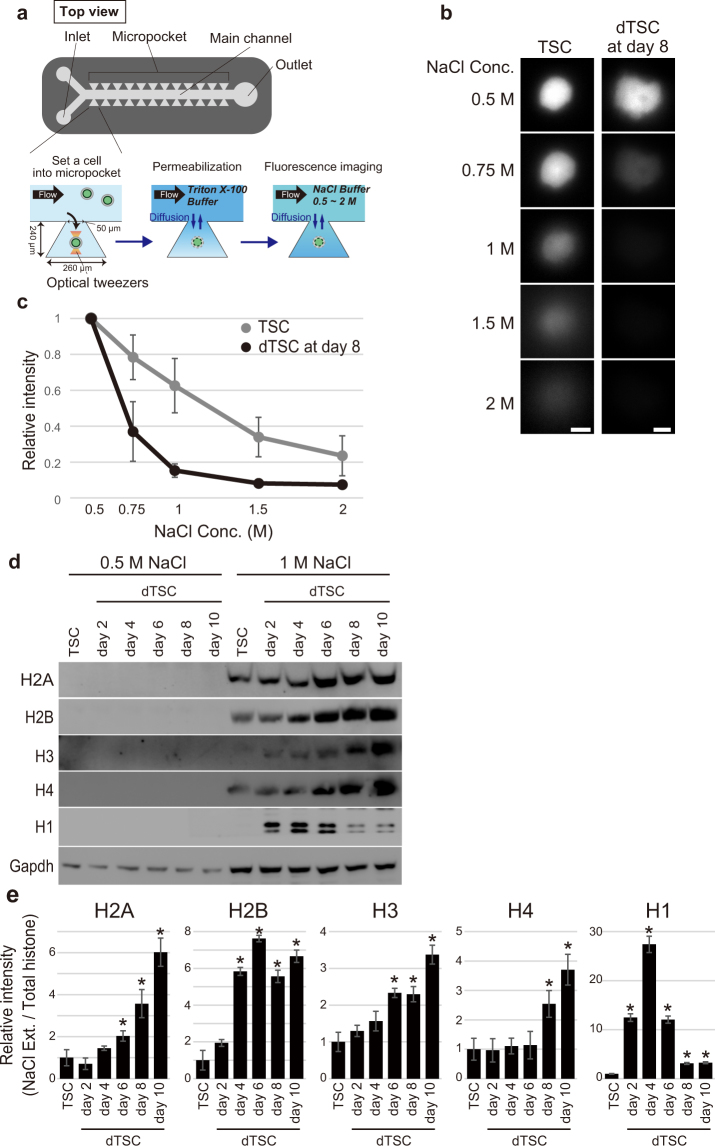
Nuclei of TGCs show decreased nucleosomal stability. (**a**–**c**) Nucleosome stability assay using a permeabilized individual cell in the microfluidic device. (**a**) Overview of the microfluidic device and experimental procedure for the nucleosome stability assay using a single cell. (**b**) Representative green fluorescence observed in H4-GFP-TSC and -dTSC at day 8 following exposure to NaCl buffer at the indicated concentrations in the micropockets. Bars = 5 μm. (**c)** Measurement of fluorescence intensity of H4-GFP in TSCs and dTSCs at day 8. Fluorescence intensities were measured using the ImageJ software, and the nuclear position in each cell was determined using the genomic DNA image obtained after GelRed staining. Values indicate the mean ± S.D. (n = 5) and are relative to the value obtained after the 0.5 M NaCl treatment, which is arbitrarily set as 1. (**d**) Nucleosome stability assay. Each sample was extracted using a buffer containing 0.5 or 1 M NaCl. Extracted proteins equivalent to 0.5 μg of genomic DNA were subjected to 15% SDS-PAGE. (**e**) Proportion of each histone in the NaCl extract to total histone. Band intensity following western blotting of 1 M NaCl-extracted histone (**d**) was calculated and normalized to that of the corresponding band in Fig. S1. The mean ± S.D. of three independent experiments are shown. *P < 0.05 versus TSC (Student’s *t*-test).

Results of the MNase digestion assay suggested that dTSCs after day 6 of differentiation had a more relaxed nucleosomal structure than TSCs. To confirm this, we evaluated the nucleosomal stability of dTSCs expressing H4-GFP in increasing concentrations of NaCl buffer by single-cell imaging using a microfluidic device (Fig. 5a). In this device, H4-GFP-TSCs or -dTSCs at day 8 of differentiation were individually placed using optical tweezers in microscale reaction chambers (hereafter referred to as micropockets), which were located along the sides of the main channel. Cells were permeabilized by introducing the Triton X-100 buffer into the main channel, and subsequently GFP fluorescence was observed after sequential exposure to 0.5 to 2 M NaCl buffer for ~20 min each (Fig. 5a). A notable decrease in the intensity of H4-GFP fluorescence in dTSCs after the exposure to 0.75 M NaCl was observed, whereas only a slight decrease was observed in TSCs. After exposure to 1 M NaCl, H4-GFP in dTSCs was barely detectable (Fig. 5b). Quantification of GFP intensity revealed a more marked decrease in dTSCs than in TSCs (Fig. 5c).

To validate the results of the nucleosomal stability assay, proteins were extracted in 0.5 or 1 M NaCl buffer from the cell pellets of TSCs and dTSCs at days 2–10 of differentiation and histones were detected using western blotting (Fig. 5d). As expected, dTSCs, particularly after day 6, showed higher solubility of core histones than TSCs in a 1 M NaCl solution. In contrast, the solubility of H1 showed an opposite trend (Fig. 5d and e).

Taken together, our results showed that as TSCs differentiated, an increase in the number of speckled foci of core histones and histone-less area in nucleus was observed. Parallely, loose nucleosome structures became apparent in dTSCs, which mostly consisted of polyploid TGCs.

### A change from canonical H3 to variant H3.3 in nucleosomes plays an important role in establishment of the unique TGC chromatin structure

**Figure 6:**
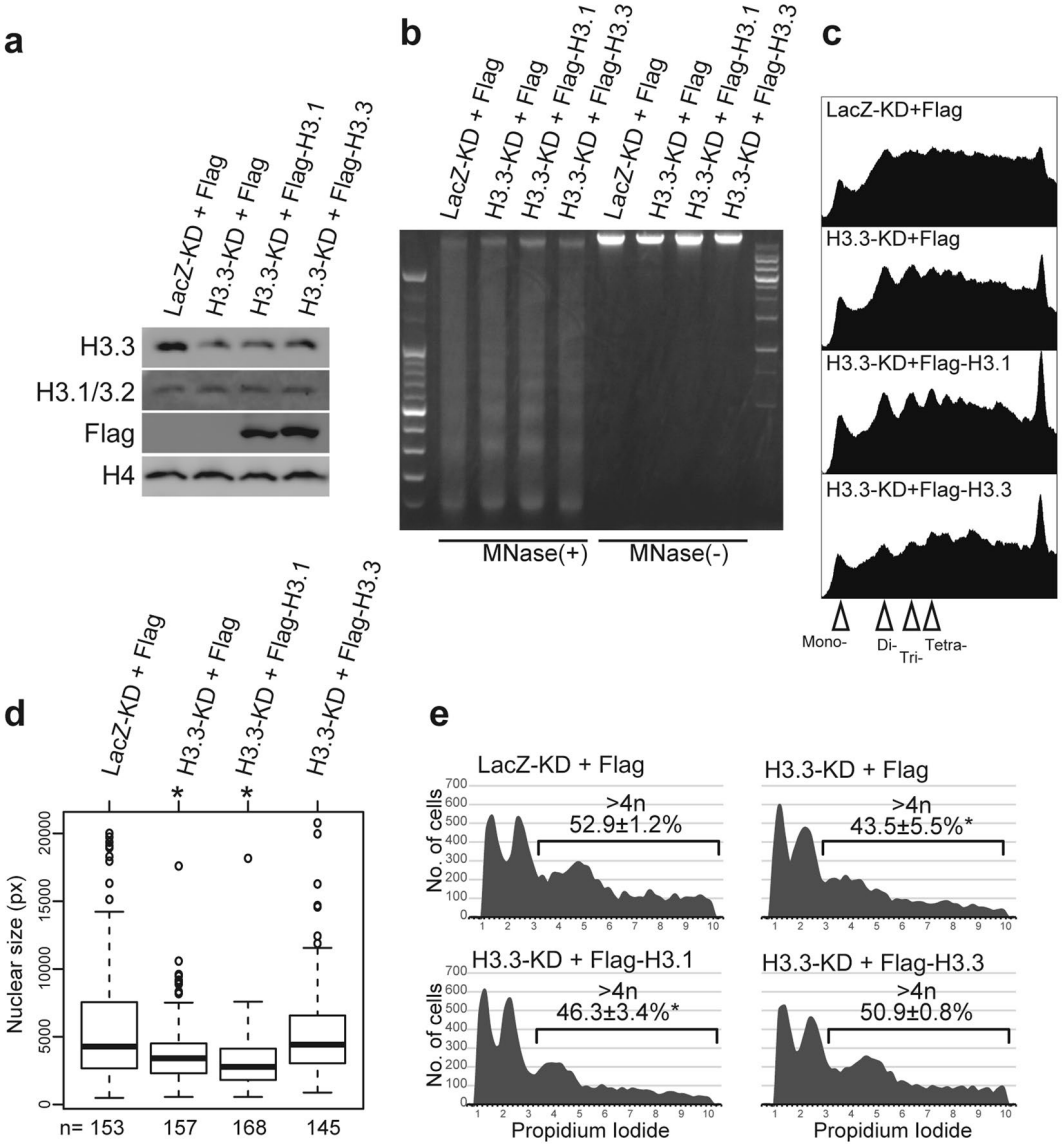
A change from H3.1/H3.2 to H3.3 is important for the establishment of loose chromatin in TGCs. (**a**) Western blotting analysis for H3.3-knockdown (KD) and H3.3-overexpressing (OE) dTSCs. The vectors for H3.3-KD and Flag-H3.3-OE were transfected at day 4 of differentiation, and all experiments were performed four days after transfection (at day 8 of differentiation). (**b**,**c**) MNase digestion analysis using chromatin from H3.3-KD and -OE dTSCs. (**b**) Gel electrophoresis image showing MNase-digested or -undigested genomic DNA. (**c**) Densitometry graph of electrophoresis image (left half) shown in (**b**) plotted using the ImageJ software. Mono, mono-nucleosome; Di, di-nucleosomes; Tri, tri-nucleosomes; Tetra-, tetra-nucleosomes. (**d**) Nuclear sizes of H3.3-KD and -OE dTSCs at day 8 of differentiation. Nuclear size was calculated from randomly selected DAPI images using the CellProfiler software. *P < 0.05 versus LacZ-KD + Flag (Wilcoxon rank-sum test). (**e**) Genomic DNA content of H3.3-KD and -OE dTSCs. DNA content was analyzed using flow cytometry of cells stained with propidium iodide. The percentages (mean ± S.D.) of nuclei with DNA content >4n are indicated in each graph. *P < 0.05 versus LacZ-KD + Flag (Student’s *t*-test).

Western blotting and immunofluorescence staining suggested a change of core histone H3 from canonical H3.1/3.2 to variant H3.3 during TGC formation. To investigate the role of H3.3 in establishment of the unique chromatin structure in dTSC nucleus, we performed H3.3 knockdown (KD) and overexpression (OE) experiments. The expression vectors for H3.3-KD or -OE were transfected into dTSCs at day 4 of differentiation. The cells were cultured for another four days and collected at day 8 of differentiation for each assay. KD and OE were confirmed using western blotting (Fig. 6a). In the MNase digestion assay, the nucleosomal bands corresponding to di- and tri-nucleosomal sizes became more conspicuous in H3.3-KD dTSCs compared to those in the control cells (LacZ-KD + Flag) (Fig. 6b and c). Rescue experiments revealed that OE of H3.3 in H3.3 KD dTSCs reversed the appearance of nucleosomal bands, whereas OE of H3.1 increased the band intensity. Moreover, enlargement of the nucleus was significantly inhibited by H3.3 KD, which was rescued by H3.3 OE, but not by H3.1 OE (Fig. 6d). H3.3-KD dTSCs also significantly decreased the number of cells containing more than 4n DNA content compared to the control cells (Fig. 6e).

These results indicated that H3.3 played an important role in formation of the unique chromatin structure and nuclear expansion in dTSCs by replacing canonical H3.

### Decrease in histone chaperone variation may be one of the key events during alteration of chromatin state from TSCs to TGCs

**Figure 7:**
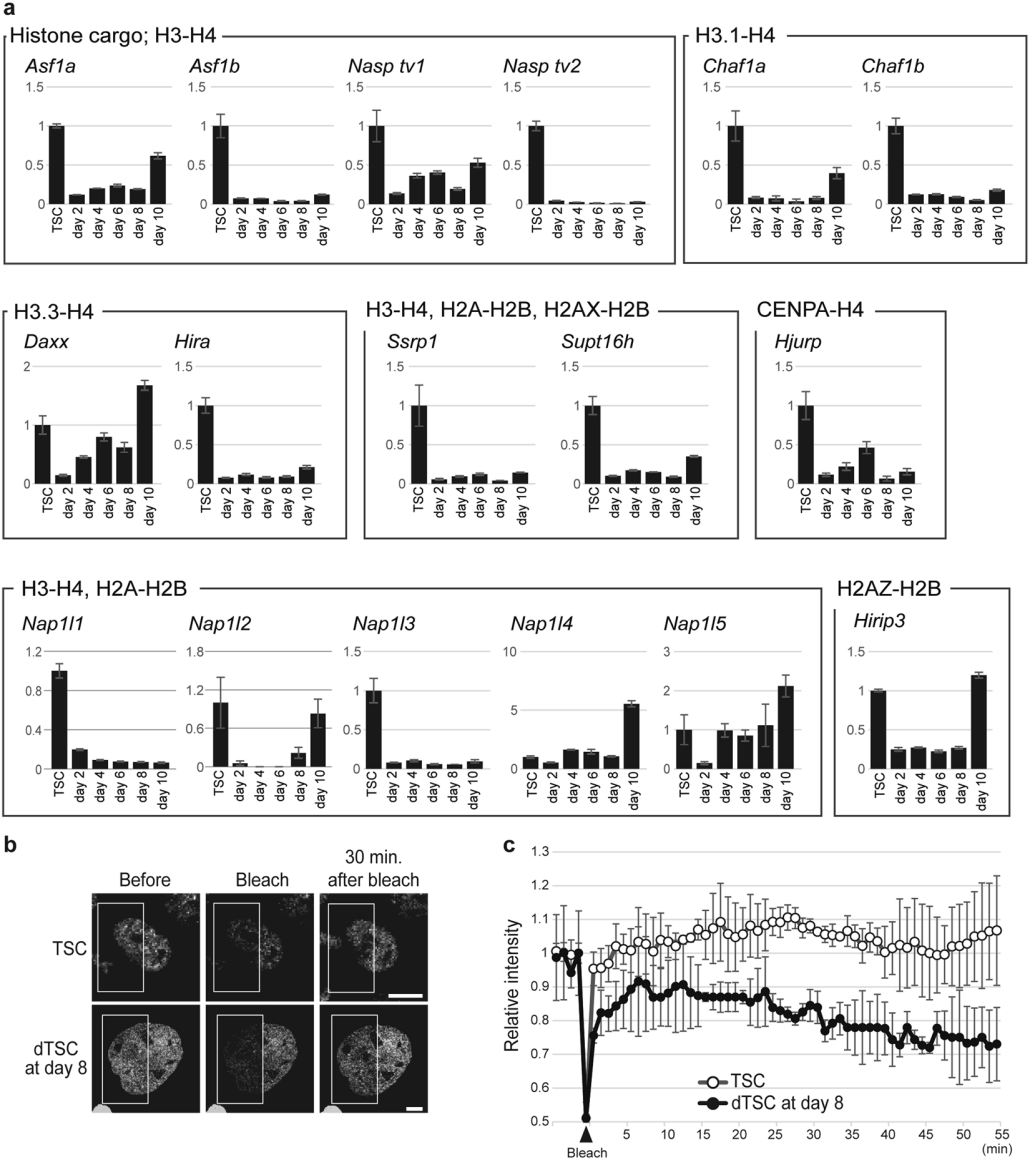
Repertoire of histone chaperones and mobility of histones are limited in dTSCs. (**a**) Expression of genes encoding histone chaperones. Graphs show the expression level of various histone chaperones determined using RT-qPCR. Values indicate the mean ± S.D. after being normalized to *Actb* expression, and are indicated relative to the levels in TSCs, which were arbitrarily set as 1. (**b**,**c**) FRAP analysis for H4-GFP-TSCs and -dTSCs at day 8. One-half of the nuclei of living H4-GFP-TSCs and -dTSCs at day 8 was bleached (white squares in **b**), and the averages of the relative fluorescence intensity (mean ± S.D.) of the bleached area up to 55-min post-bleaching are plotted (n = 10) (**c**). Bars = 5 μm.

The spatiotemporal deposition and eviction of histones by their dedicated chaperones are required to control the chromatin structure for cell-specific gene expression^10,11,24^. We next examined the expression of 17 genes encoding histone chaperones using RT-qPCR. Surprisingly, 12 out of the 17 genes showed significantly decreased expression levels upon the induction of differentiation (Fig. 7a). The expression of *Nap1l2* and *Hirip3* decreased after the induction of differentiation; however, their expression levels increased at day 10 of differentiation. The expression of *Daxx*, *Nap1l4*, and *Nap1l5* decreased at day 2 of differentiation; however, their expression was upregulated and became similar to that in dTSCs at day 4. These results suggested that these histone chaperones could affect the mobility of histones in nucleus and organization of the unique chromatin structures in dTSCs after day 6 of differentiation.

We finally examined whether there were any differences in the mobility of histones in TSCs and polyploid dTSCs by fluorescence recovery after photobleaching (FRAP) analysis using H4-GFP-TSCs and -dTSCs at day 8 of differentiation. One-half of the nucleus was bleached and fluorescence intensity was measured in the presence of cycloheximide to suppress fluorescence recovery resulting from *de novo* protein synthesis (Fig. 7b). Quantitative measurements indicated that H4-GFP was quickly recovered in TSCs (Fig. 7c). In contrast, H4-GFP in dTSCs at day 8 did not fully recover after photobleaching for 30 min. Thus, polyploid dTSCs showed less potency for histone exchange, even though they had loose nucleosomal structures.

## Discussion

**Figure 8:**
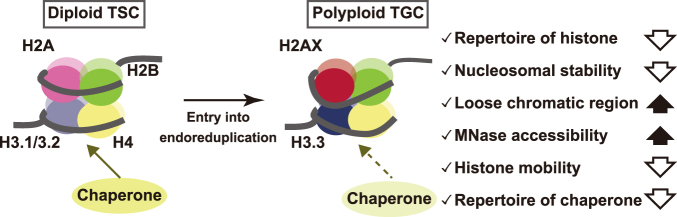
Proposed model for TGC nucleosome structure.

In the present study, we found that TGC nuclei had loose nucleosomes. Further, the variation in histone subtypes/variants decreased in TGCs, and relatively high expression of the histone variants H2AX, H2AZ, and H3.3 was observed. Previous reports indicated that these variants were required for normal embryogenesis, but their role(s) in trophoblast cells was unknown^25–27^. Our study suggested that global histone reorganization from canonical histones to these variants during TSC differentiation was associated with the formation of a unique chromatin structure for TGCs (Fig. 8).

Polytene nuclei of *Drosophila melanogaster* showed similar histone contents as the non-polytene nuclei^28^. In addition, HPLC fractionation of histones indicated that TGCs at day 6 of differentiation had a similar histone composition profile as that of TSCs^29^. These observations explained why there was no difference in the ratio of each pan-histone (H2A, H2B, H3, and H4) to total histone between polytene and non-polytene nuclei of *Drosophila melanogaster* and between TSC and TGC nuclei at day 6 of differentiation. In both studies, however, histone variants were not distinguished from canonical histones and the alteration in the expression level of each histone subtypes/variants during differentiation was not monitored. In this study, we evaluated the expression profiles of almost all histone-coding genes in details and found that the repertoire of canonical histones and histone variants reduced following the differentiation of diploid TSCs into polyploid TGCs. Although the specific functions of histone variants have been well studied^16^, the functional differences among canonical histone subtypes have not been studied. However, we recently discovered a novel canonical H2A subtype-specific histone *O*-GlcNAcylation^29^. This discovery suggested that not only the histone variants, but also the composition of the subtypes of canonical histones should be considered while studying the epigenetic organization of a cell. Expression level of each histone variant and canonical histone subtype may affect the formation of chromatin architecture and epigenetic regulation in TGCs.

Relaxed and transcriptionally active chromatin regions are thought to be maintained by H2AZ and H3.3^30^. The presence of H2AZ and H3.3 in the genome potentially correlated with actively transcribed genes, indicating that H2AZ and H3.3 were necessary for creating relaxed and transcriptionally active chromatin structures^31–33^. The present study showed that the level of H3.3 markedly increased at the expense of canonical H3 (H3.1/3.2). Meanwhile, H2AZ level was maintained until day 6 of differentiation, followed by upregulation of H2AX level from day 6 of differentiation, unlike canonical H2A levels during differentiation to TGCs even in the endoreduplication cycle. The genes encoding canonical histones are expressed at the S phase of the cell cycle^14–16^. In contrast, histone variant genes, including *H2afx*, *H2afz*, and *H3f3b*, are expressed in a cell cycle-independent manner. Since dTSCs with expanded nuclei, focused on in this study, appeared to express DNA replication markers (Fig. S4), the upregulation of H2AZ, H2AX, and H3.3 expression might not be due to cell cycle arrest but is specific to the differentiation into TGCs. Moreover, the H3.3-KD and -OE experiments in dTSCs demonstrated that H3.3 played an important role in creating loose chromatin structures. Based on the present study and previous reports on the function of H2AX, H2AZ, and H3.3, these H2A and H3 variants were potentially responsible for the formation of a loose nucleosome structure that was unique to TGCs.

Histone variants provide chromatin with temporally and spatially regulated alterations, and are required for transcriptional control of specific genes, repair of DNA damage, and DNA replication^16^. The H3.3-KD and -OE experiments using dTSCs indicated that expansion of nuclear size and polyploidization were controlled by the exchange of canonical H3 with variant H3.3. The mechanisms of polyploidization in TGCs were mainly explained by the role of cell cycle regulators such as p57Kip2 and cyclin E1/E2^34,35^. Factors associated with regulating the nuclear size of TGCs were also identified^36,37^. However, the previous studies did not focus on the influences of chromatin structures on these unique TGC phenotypes. Here, we demonstrated the importance of nucleosomal components in determining TGC phenotypes and identified H3.3 as a novel promoter of nuclear expansion and polyploidization. In the TGC genome, over- and under-replicated regions are present in a locus-dependent manner^6,7^. Considering that chromatin structures can affect the DNA replication machinery^9^, H3.3 may also play a role in establishing this non-uniform polyploid genome. In future, genome-wide analysis of H3.3 distribution in TGCs will validate this hypothesis.

As for the H2A variants, the level of both H2AZ and H2AX increased during the differentiation of TSCs. H2AX has the potential to form destabilized nucleosome structures^38^, suggesting that it plays a role in establishing and maintaining loose chromatin regions in TGCs. Phosphorylated H2AX, also called γH2AX, is well known for its role in mediating DNA damage response^39,40^. Since TGCs showed more chromatin instability than TSCs, genomic DNA might have more opportunities to become “naked” in TGCs than in TSCs, which increases the possibility of DNA damage. Nevertheless, genomic DNA must be protected to some degree for maintaining its cellular functions under any circumstances. The marked increase in H2AX level in TGCs might be explained by its role in genome protection against various environmental stresses.

In general, it is known that heterochromatin regions increase during differentiation of stem cells into terminally differentiated cells^41^. Indeed, an increase in the number of heterochromatin foci was observed after the induction of differentiation in mouse embryonic stem cells (ESCs) using several different methods, such as immunostaining of the heterochromatin protein HP1, DAPI staining^42^, and monitoring the GFP-fused methyl-CpG-binding domain^43^. In the present study, the increase in heterochromatin regions in a TGC was shown using single-cell imaging analysis. Importantly, despite an increase in heterochromatin, results of the MNase digestion assay and nucleosome stability assay indicated that the TGC had a relaxed, loose nucleosomal structure. Thus, a TGC nucleus can be regarded as having increased heterochromatin regions and regions with highly relaxed chromatin in the same genome. Isoforms of the linker histone H1 may be responsible for this unique constitution of the TGC genome. H1 is generally thought to condense chromatin structures, leading to the repression of gene expression^44,45^. In this study, the nucleosome stability assay showed a marked decrease in H1 solubility in NaCl solution after day 8 of differentiation, in contrast to the solubility of core histones. In the terminally differentiated dTSCs, the expression levels of two H1 genes, namely *Hist1h1c* (encoding H1.2) and *H1f0* (encoding H1.0), increased. Therefore, we hypothesize that the heterochromatin regions observed in TGC nuclei were formed and maintained mainly by the linker histones H1.2 and H1.0. Genome-wide distribution analysis and KD of these H1 isoforms can help in validating this hypothesis.

Deposition and replacement of histones are important to maintain and alter the overall chromatin structure, which is mainly controlled by histone chaperones^10,11^. In TGCs, the diversity of histone chaperones as well as the histone variety decreased during differentiation. This decrease in diversity may be related to the hindered mobility of histones in TGCs than in TSCs, as shown by FRAP analysis. Our results also indicated that the expression of *Daxx*, a H3.3-specific chaperone^46^, increased and remained high during the differentiation process, along with the existence of H3.3 in the nucleus of TGCs. Gene expression of H2AX- and H2AZ-specific chaperones, members of the FACT complex (*Ssrp1* and *Supt16h*) and *Hirip3*^47,48^, respectively, significantly decreased after the induction of differentiation. Thus, it seemed unlikely that these H2A variants were dependent on these dedicated chaperones for being incorporated into the TGC nucleosomes at least by day 8 of differentiation. Developmental process and cell fate decisions are coupled to chromatin remodeling and concomitant replacement of histone molecules by specific histone chaperones^24^. The limited usage of histone chaperones in TGCs may support not only the establishment/maintenance of the unique chromatin structures but also TGC differentiation.

An increase in the amount of genomic DNA is associated with the enlargement of nucleus and cell body during differentiation, which is a common event in polyploid cells^49^. However, an increase in the DNA content is even more intense than the expansion of nuclear size in some highly polyploid cells, including TGCs. Furthermore, it is known that unlike diploid cells, transcript levels are not proportional to the amount of DNA or gene copy number in polyploid cells^50^. The present study, which analyzed nucleosomal contents and chromatin structures in TGCs, indicated that a unique combination of histone variants in TGCs establishes the characteristic chromatin structure, which enables an enormous amount of DNA to be precisely packaged in the nucleus and results in a unique gene expression system.

## Methods

### Reagents

All reagents were purchased from Wako Pure Chemical, unless otherwise mentioned. Antibodies used in this study are listed in Table S1. Primer sequences are shown in Table S2.

### Cell culture

Mouse trophoblast stem cells (B6TS4 line) derived from a C57BL/6 N blastocyst in our laboratory were cultured and maintained in an undifferentiated state in the 70CM + F4H medium^51^. Differentiation was induced by withdrawal of FGF4 and heparin. The cells were harvested using 0.05% trypsin–1 mM EDTA in phosphate buffered saline (PBS) without divalent cations [PBS(−)], washed with PBS(−), collected in 1.5 or 2 mL tubes, snap frozen in liquid nitrogen, and stored at −80 °C until further use (DNA/RNA/protein extraction).

### Collection of parietal TGCs

The parietal TGCs on Reichert’s membrane were harvested from the concepti of ICR mice (Charles River, Japan) on day 9 of pregnancy, according to the method described in a previous report^6^. The embryos and decidual cells were harvested and used as control diploid cells. The tissues were rinsed in PBS(−) to remove blood, collected in 1.5 mL tubes, snap frozen in liquid nitrogen, and stored at −80 °C until use. Immunofluorescence staining was performed by placing the Reichert’s membrane samples, which contain parietal TGCs, on amino silane (APS)-coated glass slides (Matsunami, Japan) using fine tweezers.

### RT-qPCR using the BioMark system

Total RNA was isolated from cells using the TRIzol reagent (Invitrogen), according to the manufacturer’s instructions. cDNA was synthesized from 1 μg total RNA using either of the two primers, namely random hexamers or oligo(dT)_20_, and the SuperScript III First-Strand Synthesis System (Invitrogen). RT-qPCR was performed using a high-throughput gene expression platform based on microfluidic dynamic arrays (BioMark, Fluidigm)^52^. Data were processed by automatic global threshold setting with the same threshold value for all assays and linear baseline correction using the BioMark Real-Time PCR Analysis software (Fluidigm). Results of RT-qPCR for histone-coding genes by BioMark were visualized as a heatmap using the MeV software^53^.

### Histone purification, nuclear and cytoplasmic protein extraction

Histone fractions were collected from two 100-mm dishes containing confluent cells using the Histone Purification Mini kit (Active Motif), according to the manufacturer’s instructions. Genomic DNA was also purified from an aliquot of cell pellets used for histone purification. For western blotting, the amount of total histone was normalized by genomic DNA, namely total histone equivalent to 0.5 μg of genomic DNA were loaded into each well of SDS-PAGE.

Nuclear and cytoplasmic proteins were extracted using the LysoPure Nuclear and Cytoplasmic Extractor kit, according to the manufacturer’s instructions. The amount of cytoplasmic and nuclear protein was estimated using the BCA assay.

### Western blotting

Proteins were fractionated using 15% SDS-PAGE, blotted onto Millipore transfer membranes (Merck Millipore), and incubated at 4 °C with 1 μg/mL of primary antibodies. Protein bands were detected using a secondary antibody (1:4,000) conjugated to horseradish peroxidase (Jackson ImmunoResearch), and ImmunoStar basic or ImmunoStar LD as the substrate. Amount of the histone fraction in each sample was normalized to the amount of genomic DNA. All uncropped images are presented in Fig. S5.

### Immunofluorescence staining

Cells cultured in 4-well plates and parietal TGCs on APS–coated slide glass were fixed using 4% paraformaldehyde for 20 min at room temperature, permeabilized using 0.2% Triton X-100 for 30 min at room temperature, followed by blocking with 5% BSA (Rockland) and 0.1% Tween 20 in PBS(−) overnight at 4 °C, and incubation with 1 μg/mL of the primary antibody overnight at 4 °C. The secondary antibody (1:1,000) was added and incubated for 1 h at room temperature. Nuclei were stained using DAPI (1 μg/mL; Dojindo). Images were captured using the LSM 700 laser scanning confocal microscope (Zeiss).

### Construction of expression vectors

Mouse full-length *Hist1h4i* (encoding H4) was obtained from TSC cDNA using PCR amplification. The cDNA was cloned into the pGEM-T Easy vector (Promega), and the resulting constructs were confirmed using BigDye sequencing (Invitrogen). The cloned cDNA was inserted into pEGFP-N3 (Clontech) at the NheI and EcoRI sites to generate the expression vectors for H4-GFP.

Mouse full-length *Hist1h3a* (encoding H3.1) and *H3f3a* (encoding H3.3) were obtained using cDNA from mouse ESCs (with a 3× Flag-tag inserted) by two PCR amplifications and subsequently ligated into the pENTR/D-TOPO vector (Life Technologies). The appropriate inserts were confirmed using BigDye sequencing. 3× Flag-fused genes were subcloned into the pCAG-DEST-PGK-Puromycin-IRES-VENUS-pA vector^54^ using Gateway LR Clonase (Life Technologies).

For H3.3 knockdown, two specific miRNA sequences targeting the respective UTR regions of *H3f3a* and *H3f3b* were cloned into the pcDNA 6.2-GW/EmGFP-miR vector (Thermo Fisher Scientific). Similarly, a sequence targeting the LacZ-encoding mRNA was cloned and denoted control-miR.

Plasmids were purified using the Quantum Prep Plasmid Midi Prep kit (BIO-RAD), followed by phenol:chloroform:isoamyl alcohol (PCI) extraction and ethanol precipitation. For knockdown and overexpression experiments using dTSCs, 10 μg plasmids were transfected into dTSCs placed in a 100-mm dish at day 4 of differentiation using 20 μL jetPRIME reagents (Polyplus)^51^. About 24–96-h post-transfection, dTSCs were selected using 2 µg/mL blasticidin and 1.5 µg/mL puromycin.

### Live cell imaging for H4-GFP

To establish a TSC line stably expressing GFP-fused histone H4 (H4-GFP-TSC), TSCs were cultured in 6-well plates to ~50% confluence and subsequently transfected with 3 μg plasmid and 9 μL jetPRIME reagent per well. Twenty-four hours after transfection, cells were replated and cultured for one week in a 100-mm dish in the presence of 1 mg/mL G418. Individual G418-resistant colonies were picked and transferred to each well of a 96-well plate. Cells that expressed the fusion proteins were expanded, collected, suspended in Cell Culture Freezing Media (CellBanker 1; Takara), frozen in liquid nitrogen, and stored at −80 °C until further use.

Imaging for GFP was performed using a confocal microscope (CV1000; Yokogawa), equipped with a heated stage and cover filled with humidified 5% CO_2_ and 95% air. H4-GFP-TSCs were seeded onto a 35-mm glass-bottom dish and cultured in TS medium without FGF4/heparin to induce differentiation. Two days after the induction of differentiation, confocal images were obtained at 30-min intervals for 96 h using a 100× objective lens.

### MNase digestion analysis

MNase digestion analysis for unfixed cells was performed using the EZ Nucleosomal DNA Prep kit (Zymo Research). Briefly, cytoplasm was removed using the Nuclei Prep buffer and the remaining chromatin was washed twice with the Atlantis Digestion buffer. Chromatin (equivalent to 1 μg DNA) was digested using 0.0014 U MNase in the Atlantis MN Digestion buffer.

MNase digestion analysis for fixed cells was performed using the ChIP IT Express Enzymatic kit (Active Motif). Cells were fixed using 1% formalin for 15 min at room temperature, and cytoplasm was removed using the 1× lysis buffer supplemented with 1 mM phenylmethylsulfonyl fluoride (PMSF) and 1× protease inhibitor cocktail. Chromatin (equivalent to 1 μg DNA) was digested using 0.0020 U MNase in the digestion buffer. Cross-linking of DNA was reversed by incubation in 5 M NaCl/lysis buffer at 65 °C overnight.

Digested DNA was purified using PCI extraction and ethanol precipitation, and electrophoresed on 1.2% agarose gel.

### Nucleosome stability assay using a microfluidic device

The microfluidic device was fabricated as described previously^55^ and placed on an XY motorized stage (Sigma Koki) mounted on an inverted optical microscope (IX-71; Olympus). H4-GFP-TSCs or H4-GFP-dTSCs at day 8 were resuspended in isotonic buffer (300 mM sorbitol and 5 μM Hoechst 33342), introduced into the main channel from the inlet and transported and placed in the micropockets one-by-one using optical tweezers (Fig. 5a). The optical tweezers were operated as described previously^55^. Cells were exposed to the Triton X-100 buffer [1% Triton X-100, 10 mM Tris-HCl (pH 8.0), 1 mM EDTA, 10 mM DTT, and GelRed (1:50,000)] in the micropockets for 20 min to permeabilize them. The solution conditions in micropockets could be rapidly altered by diffusion of solutes, when isotonic buffer in the main channel was replaced with the Triton X-100 buffer and the newly introduced solution was kept flowing^55^. After permeabilization, the cells were exposed to NaCl buffer [0.5/0.75/1/1.5/2 M NaCl, 10 mM Tris-HCl (pH 8.0), 10 mM DTT, and GelRed (1:50,000)] by replacing solutions in the main channel with increasing NaCl concentration in a stepwise manner. Here, free histone proteins which dissociate from chromatin would diffuse out to the main channel, while nuclei containing chromatin/DNA will remain inside the micropockets due to their small diffusion coefficients. After the cells were exposed to each concentration of NaCl buffer for approximately 20 min, fluorescence images of GFP (histone H4) and GelRed (genomic DNA) were obtained using a high-sensitivity video camera (EM-CCD camera, C9100-13; Hamamatsu Photonics), and fluorescent intensity was quantified using the ImageJ software (https://imagej.nih.gov/ij/).

### Nucleosome stability assay using frozen cell pellets

Frozen cell pellets were thawed on ice, resuspended in buffer A [20 mM HEPES (pH 7.9), 0.5 mM DTT, 1 mM PMSF, 1.5 mM MgCl_2_, and 0.1% Triton X-100] containing 0.5 or 1 mM NaCl, and incubated for 40 min at 4 °C with constant agitation. Cell lysates were centrifuged at 20,000 × *g* at 4 °C for 30 min. The supernatant was transferred into Amicon Columns (Millipore) and concentrated by centrifugation at 14,000 × *g* at 4 °C for 10 min. The extracted liquid was collected in Protein LoBind tubes by centrifugation at 1,000 × *g* at 4 °C for 2 min with reverted columns and subjected to western blotting.

Genomic DNA was also purified from an aliquot of cell pellets used for protein extraction. Cells were dissolved in lysis solution [10 mM Tris-HCl (pH 8.0), 5 mM EDTA, 200 mM NaCl, 0.2% SDS, and 200 μg/mL proteinase K] at 55 °C for 30 min. DNA was extracted with PCI, incubated with 5 μg/mL RNase A for 30 min, and re-extracted with PCI. DNA was precipitated using ethanol and dissolved in Tris-EDTA (TE) buffer (pH 8.0).

### Measurement of DNA content

Cells were fixed in 70% ethanol and stained using 20 μg/mL propidium iodide and 100 μg/mL RNase (Sigma). Fluorescence intensity of each cell was analyzed using the BD FACSVerse flow cytometer (BD Biosciences).

### Measurement of nuclear size

Cells cultured in 4-well plates were fixed using 4% paraformaldehyde for 20 min at room temperature and the nuclei were stained with DAPI. For measurement of the size of the nucleus in dTSCs, fluorescence images after DAPI staining were processed using the CellProfiler software as described previously^56^.

### FRAP analysis

FRAP was performed using a confocal microscope (FV-3000; Olympus), equipped with a heated stage and cover filled with humidified 5% CO_2_ and 95% air. A confocal image of a field containing approximately five nuclei was obtained using a 60 × lens (512 × 512 pixels, 2 μs/pixel scan speed, 2 AU pinhole, 500–600 nm variable filter, and 0.33% transmission of 488-nm Ar laser). Thereafter, one-half of each nucleus was photobleached using 10% transmission of the 488-nm laser and images were collected using the original setting at 1-min intervals for 60 min. Fluorescence intensities of the bleached, unbleached, and background areas were measured using the FV31S-SW software (Olympus). After background subtraction, relative intensity of the bleached area to the unbleached area was determined and normalized to the initial value before bleaching.

### Statistical analyses

Student’s *t*-test was performed for comparison of western blotting (Figs 2a,b, 3b,c and 5e) and FACS analysis (Fig. 6e), and the Wilcoxon rank-sum test was used for comparing nuclear sizes (Fig. 6d). A *P*-value < 0.05 was considered to be statistically significant.

### Ethics statement

All experiments using mice were carried out according to the institutional guidelines for the care and use of laboratory animals. The procedures used were approved by the Committee for Life Science Research Ethics and Safety, Graduate School of Agriculture and Life Sciences, The University of Tokyo. The mice were humanely euthanized by cervical dislocation to minimize suffering.

### Data availability

All data generated or analyzed during this study are included in this published article and its Supplementary Information files.

## Electronic supplementary material

Supplementary information

Supplementary movie S1a

Supplementary movie S1b
