# Supplementary material for: Nucleosomes of polyploid trophoblast giant cells mostly consist of histone variants and form a loose chromatin structure
Source: Sci Rep. 2018 Apr 11;8:5811. doi: 10.1038/s41598-018-23832-2 (PMC5895725; doi:10.1038/s41598-018-23832-2)
Supplement: Supplementary file 1 — Supplementary information [file 41598_2018_23832_MOESM1_ESM.pdf]

Supplementary information

TITLE

Nucleosomes of polyploid trophoblast giant cells mostly consist of histone variants and form a loose chromatin structure

AUTHORS

Koji Hayakawa[1, *, **], Kanae Terada[1, *], Tomohiro Takahashi[2], Hidehiro Oana[2], Masao Washizu[2, 3], Satoshi Tanaka[1, **]

[1] Laboratory of Cellular Biochemistry, Department of Animal Resource Sciences /Veterinary Medical Sciences, The University of Tokyo, Tokyo, Japan
[2] Department of Mechanical Engineering, The University of Tokyo, Tokyo, Japan
[3] Department of Bioengineering, The University of Tokyo, Tokyo, Japan

* These authors contributed equally to this work
** To whom correspondence should be addressed: Koji Hayakawa and Satoshi Tanaka. Laboratory of Cellular Biochemistry, Department of Animal Resource Sciences/ Veterinary Medical Sciences, The University of Tokyo, Tokyo 113-8657, Japan
TEL/FAX: +81-3-5841-5472/+81-3-5841-8189. Email: akojih@mail.ecc.u-tokyo.ac.jp (K.H.) and asatoshi@mail.ecc.u-tokyo.ac.jp (S.T.)

Table S1 and S2
Figure S1-S5
Movie S1 (legend only)

Table S1 Antibody list

Target	Clonality	Company	Cat. No.
H2A	Rabbit Poly	Abcam	ab15653
H2B	Rabbit Poly	Abcam	ab1790
H3	Mouse Mono	MABI	MABI 0301
H4	Mouse Mono	MABI	MABI0400
H2A.X	Rabbit Poly	Abcam	ab11175
H2A.Z	Rabbit Mono	Abcam	ab150402
H3.1/3.2 (D4F2)	Mouse Mono	Cosmo Bio	CE-039B
H3.3 (4H2D7)	Rat Mono	Cosmo Bio	CE-040B
H1 (AE-4)	Mouse Mono	Santa Cruz	sc-8030
PL-I	Goat poly	Santa Cruz	sc-34713
PL-I	Mouse mono	Santa Cruz	sc-376436
FLAG (M2)	Mouse mono	Sigma	F1804
GAPDH	Mouse mono	IMGENEX	IMG-5019A-1
DESMIN	Goat poly	Santa Cruz	sc-7559
PCNA	Mouse mono	Santa Cruz	sc-56
Ki-67	Mouse mono	Dako	M7240
Anti-goat IgG (HRP-conjugated)	Donkey poly	Jackson ImmunoResearch	705-035-147
Anti-mouse IgG (HRP-conjugated)	Goat poly	Jackson ImmunoResearch	115-035-003
Anti-rabbit IgG (HRP-conjugated)	Goat poly	Jackson ImmunoResearch	111-035-003
Anti-rat IgG (HRP-conjugated)	Goat poly	Jackson ImmunoResearch	112-035-167
Anti-mouse IgG (Alexa Fluor 594-conjugated)	Goat poly	Invitrogen	A11005
Anti-rabbit IgG (Alexa Fluor 488-conjugated)	Goat poly	Invitrogen	A11008
Anti- rabbit IgG (Alexa Fluor 594-conjugated)	Goat poly	Invitrogen	A11037
Anti-rat IgG (Alexa Fluor 488-conjugated)	Goat poly	Invitrogen	A11007
Ab-10 Rapid HiLyte Fluor 555 Labeling Kit	This kit was used for labeling pan-H2A antibody in IF assay.	Dojindo	LK35

Table S2 Primer list

For RT-qPCR

Gene	Forward	Reverse	Universal Probe ID
H2afj	CAGGAGAAGCGGACCTAGTG	CTGGGGAGTTAGGCAAAGC	#110
H2afv	GCGGTGATGAAGAGTTGGAT	CCCCTTCTTTCCAATCAGAGA	#49
H2afx	AAGCCGGTGAATCCCTGT	AGCTGCAAAAGTTCCAGTTCA	#106
H2afy	TGACATTGACCTTAAAGATGACCT	CCAGAACAGCTTCTACAAACTCC	#47
H2afy2	AGTGGGGCTCCGACAAAT	TGCAGACAGGCAGTTTTTGA	#11
H2afz	CTCAGGACTCTAAATATTCCTAACAGC	TGTTTTTCACAGAGATACAGTCCAC	#47
Hist1h2aa	GTATCTGGCGGCAGTGCTA	TAATGCGCGTCTTCTTGTTG	#1
Hist1h2ab	GTCCTGCCCAACATCCAG	CAGTACAGACAATCGCCAGATTA	#74
Hist1h2ac	GAAATTAGAAGAGCACAGGCTGAT	AGCATGCAGTGGTGCCTAC	#46
Hist1h2ad	GTCCTGCCCAACATCCAG	TTATTTCCCCTTGGCCTTG	#74
Hist1h2af	GTCCTGCCCAACATCCAG	CTTTCCCTTGGGCTTATGG	#74
Hist1h2ag	GTCCTGCCCAACATCCAG	CAACAGTGCTTTGTATAAAGGGTTT	#74
Hist1h2ak	TGGCAGCCGTGCTAGAGTA	TAATGCGCGTCTTCTTGTTG	#1
Hist1h2an	ACGACGAGGAGCTCAACAA	CTTCTTCGGCAGCAGTACG	#107
Hist1h2ao	GTCCTGCCCAACATCCAG	TTTTCCCTTGGCCTTGTG	#74
Hist2h2aa1	GCCCGCGTCTCTGTGATA	AGACCGGCTACCGTGACA	#21
Hist2h2aa2	GTCCTGCCCAACATCCAG	CTTGCCCTTCGCCTTATG	#74
Hist2h2ab	CGGTGCTGGAGTACCTAACG	TTCCTCACAGCTAGTTGCAGAT	#1
Hist2h2ac	AAAGTGACGATCGCACAGG	CTTGTGGCTCTCGGTCTTCT	#74
Hist3h2a	TGGAGGGAGGTGTACTAGGG	TTGGGTAGTGGTTGTGCATTT	#66
Hist1h2ba	ACAAGTGCACCCCGACAC	TCGAAGATGTCTGTCACAAAGG	#25
Hist1h2bb	GTCCCGGGAGATCCAGAC	TGTACTTGGTGACGGCCTTA	#40
Hist1h2bc	ACACCAGCTCCAAGTGATCC	CCAGCACTGTTGAGTGGTACA	#68
Hist1h2be	GAACTAAAGGCCCAGTTCCA	TCAGAAGGGAGCCATGGTAG	#42
Hist1h2bf	ACAAGGTGCTGAAGCAAGTG	GCTCGAAGATGTCGTTCACA	#105
Hist1h2bg	TTGTTGCTTGTTTCTACCATGC	GCCTTCTTGGAGCCCTTCT	#51
Hist1h2bh	GTCCCGGGAGATCCAGAC	TTGGTGACAGCCTTGGTG	#40
Hist1h2bj	CCGACACCGGTATCTCCTC	GCGCTCGAAGATGTCGTT	#105
Hist1h2bk	GTCCCGGGAGATCCAGAC	GGCGCTAGTATACTTGGTGACA	#40
Hist1h2bl	GTCCCGGGAGATCCAGAC	TTGGTGACTGCCTTGGTG	#40
Hist1h2bm	GTCCCGGGAGATCCAGAC	GTACTTGGTGACCGCCTTG	#40

Hist1h2bn	AGCGCTCGACCATCACAT	CTTGGTGACAGCCTTGGTG	#40
Hist2h2bb	TTCCCGGGAGATCCAGAC	CTTCCGACACAGCGTGTTTA	#74
Hist3h2bb	ATGCCTGATCCATCCAAATC	TTGGTGACCGCCTTTTTG	#19
H3f3a	GCCATCTTTCAATTGTGTTCG	AGCCATGGTAAGGACACCTC	#19
H3f3b	CGATTGCGGCTCTTGTTC	CTTGGTTCGGGCCATTTT	#11
Hist1h3a	ACGGAATTAACCAAAGGCTCT	GGAGTAAATTACAGCCATTTTACTTGA	#21
Hist1h3b	CAGGACTTCAAGACCGACCT	TGTCCTCAAACAGACCCACA	#21
Hist1h3c	AAGCGTGTCACCATCATGC	GCATAAAAACCATTATGCCCTCT	#42
Hist1h3g	CCAAGCGTGTCACCATCA	AACAGAAACCCTTAAGCCCTCT	#42
Hist1h3h	AAGCGTGTCACCATCATGC	CCTTTGAAAAACACAGAAAGACG	#42
Hist1h3i	AAGCGTGTCACCATCATGC	GTAGGGGAATTACGCCCTCT	#42
Hist2h3c1	AGCGCGGGAGTTTCAAGT	CCTTTATAGGCGCAGTCTTCC	#94
Hist1h4a	CGCCACCGTAAGGTTCTC	CGTAGATGAGGCCGGAGAT	#9
Hist1h4b	TAAGCGCCATCGCAAAGT	CGTAGATGAGGCCGGAGAT	#9
Hist1h4c	CGTCACCTACACGGAGCAC	CGCCGAATCCGTAGAGAG	#81
Hist1h4d	AGTTCTGCGCGACAACATC	GGAGATGCGCTTCACTCCT	#70
Hist1h4f	GCCACCGTAAGGTTCTTCG	GGAGATGCGCTTCACTCCT	#70
Hist1h4h	CGTCACCTACACGGAGCAC	GTCAGAAGCAAGATTCGCTTAAC	#81
Hist1h4i	CAGTGTTCTTTTTGTCCTGGAA	TAATCCCAACACTCGGGAAG	#64
Hist1h4j	AAAGGGCTCGGTAAAGGTG	CGTAGATGAGGCCGGAGAT	#70
Hist1h4k	CAAAGTGCTGCGCGATAAC	CGTAGATGAGGCCGGAGAT	#9
Hist1h4m	AACATCCAGGGCATCACG	GGAGATGCGCTTCACTCCT	#70
Hist1h1a	AGAAGAACAACAGCCGCATC	TGCACCAGTGTGCCTTTATT	#45
Hist1h1b	TCCAAAGAAAGCGAAGAAGC	TCTTAGGACTCTTGGCAACTTTCT	#19
Hist1h1c	GCGTGTCCCACCTAGACTTG	GTGTTCCAGCCCATAGCACT	#26
Hist1h1d	CGTCTACGTTTCAATCTAGCATTTC	TGAGGCTCGAATACAAGATCC	#19
Hist1h1e	GAACAAACCTTAGTGGGCTGA	GTTAACACCATTGCGCACAC	#75
Hist1h1t	CCAGCTCTTGACCATGTCG	CAGGAGCTGGAACAAGGGTA	#92
H1f0	TGGAGACCTGGTTGCTTTTT	TTTGCCCCTTTAGACAATGG	#29
H1fx	GCTGCGTTCTGTTTGCTTTA	GTTGCCAAGGGTGAGAGC	#81
Asf1a	TGTTTTAGTGGGTCCCGTTC	CTGCATTCGGTGCATCAG	#4
Asf1b	TCAGCTTCGAATGCAGTGAG	TGAGCCCACATAAATGATCTTC	#85
Chaf1a	CCGTGGATTGCAAAGACAG	GCAAGCGGGCTTGTATTAAC	#48
Chaf1b	TCCGAAGACTCAGCTTTACTCC	TCACATTCTCACCAGATTCCA	#19
Daxx	GAAGATGAAGATGAGGATCTAGAACAG	TGGGACTCTCATTTCCTTCATTAT	#4

Hira	GCTGGACTGGCAGCTAGAGA	ACCAACTAAGCCGGAGAACA	#9
Nasp tv1	AAACAGAAGATGAATCTTTGGTAGAAA	TTGGTGTTTCTTCAGCCTTGT	#6
Nasp tv2	GGGTGGAAGAGAAGACATGG	TGCTTTGAAGTCGGTTCAACT	#1
Hjurp	TCCAACAGGAAGCCACAGA	ACAGCCAGGTGGCACTTTT	#40
Supt16h	AAGTGCCATTTAGGGACCTG	GGTGGCCACTCTGTAGCATT	#16
Ssrp1	CAATGACAGCAGCGACGA	CAGAGGCATTGCTGTCAAAC	#2
Nap1l1	CCCCCACTCCCACTTCTT	CCCATTGGACATGCAAACTT	#2
Nap1l2	GTAATCATACTGCGGCACCA	CAGCTGCTCCAGCCACTC	#58
Nap1l3	AATAGGTGTACGGGGCTAACAG	CACCAGGTTCTGTGACCATTT	#29
Nap1l4	ACAACGTCTCTCACACTCCTTCT	TGAACTTCCTCGTAGAACTTAGCTT	#1
Nap1l5	TCCCAACTTCGCCAAGAA	TAAAAGGGGCAGGGGATTTA	#99
Hirip3	TAAGATGGCCTTGGGGTTC	AAACAACAGTCCACTCAAATGC	#55
Actb	GGATGCAGAAGGAGATTACTGC	CCACCGATCCACACAGAGTA	#63

For construction of over-expression vectors

Primer name	Sequence
NheI_Koz_Hist1h4i_F	GCTAGCACCATGTCTGGTCGTGGCAAAGGAGGAAAAG
EcoRI_Hist1h4i_R	GAATTCCCGCCGAACCCGTACAGAGTACGGC
3xFlag_F2	CACCATGGACTACAAAGACCATGACGGTGATTATAAAGATCATGATATCGATTACAAGG
3xFlag_R	CTACTTGTCATCGTCATC
Flag-Hist1h3a_F	ATCATGATATCGATTACAAGGATGACGATGACAAGATGGCTCGTACTAAGCAGACCGCT
Hist1h3a_R	TTACGCCCTCTCCCCGCGGATGCGGCGG
Flag-H3f3a_F	ATCATGATATCGATTACAAGGATGACGATGACAAGATGGCTCGTACAAAGCAGACTGCC
H3f3a_R	TTAAGCACGTTCTCCGCGTATGCGGCGT

For construction of knockdown vectors

Primer name	Sequence
H3f3a_miR_27_F	TGCTGATTGAAAGATGGCTGACACCGGTTTTGGCCACTGACTGACCGGTGTCACATCTTTCAAT
H3f3a_miR_27_R	CCTGATTGAAAGATGTGACACCGGTCAGTCAGTGGCCAAAACCGGTGTCAGCCATCTTTCAATC
H3f3b_miR_1127_F	TGCTGAACTGGACTAAATTACACACAGTTTTGGCCACTGACTGACTGTGTGTATTAGTCCAGTT
H3f3b_miR_1127_R	CCTGAACTGGACTAATACACACAGTCAGTCAGTGGCCAAAACTGTGTGTAATTTAGTCCAGTTC
miR_LacZ_top	TGCTGAAATCGCTGATTTGTGTAGTCGTTTTGGCCACTGACTGACGACTACACATCAGCGATTT
miR_LacZ_bottom	CCTGAAATCGCTGATGTGTAGTCGTCAGTCAGTGGCCAAAACGACTACACAAATCAGCGATTTC

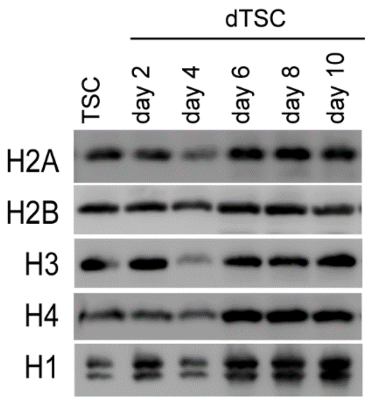

Supplementary figure S1 Representative western bolting images of toal core histones and linker histone H1 in TSCs and dTSCs.

Western blotting analyses with pan antibodies against each core histone were performed using total histones equivalent to 0.5 μg of genomic DNA.

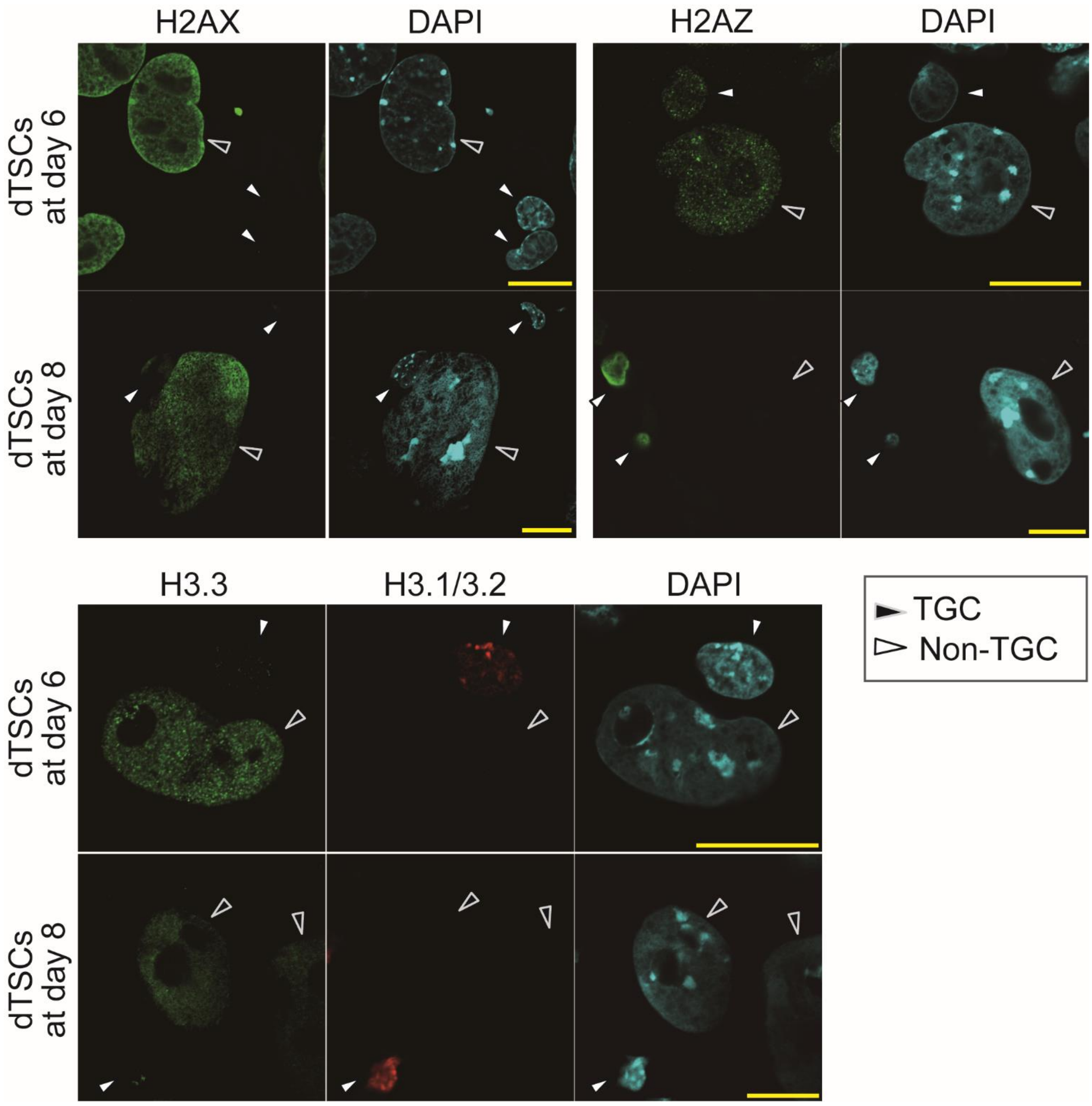

Supplementary figure S2 Immunofluorescence staining of H2AX, H2AZ, H3.1/3.2, and H3.3 in dTSCs at days 6 and 8 of differentiation.

Black and white arrowheads indicate dTSCs with a large nucleus (putative TGCs) and dTSCs with a small nucleus (non-TGCs), respectively. Bars = 20 µm.

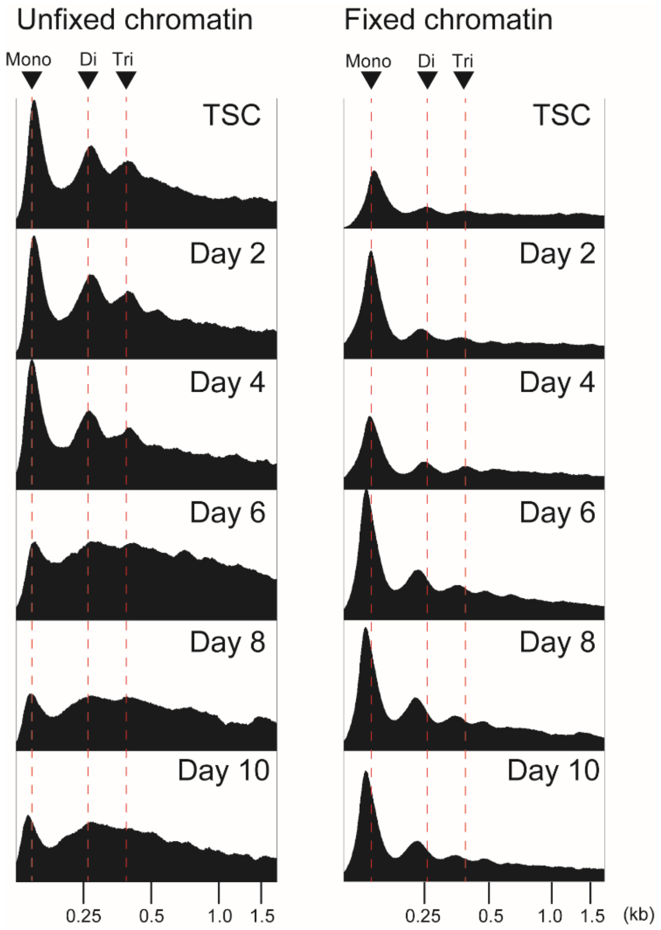

Supplementary figure S3 Densitometric graphs of electrophoresis image shown in Fig. 4b and 4c.

The images were analyzed and graphs were plotted using Image J software. Red broken line indicates the position of mono-, di-, or tri-nucleosomes in TSC. Mono, mono-nucleosome; Di, di-nucleosomes; and Tri, tri-nucleosomes.

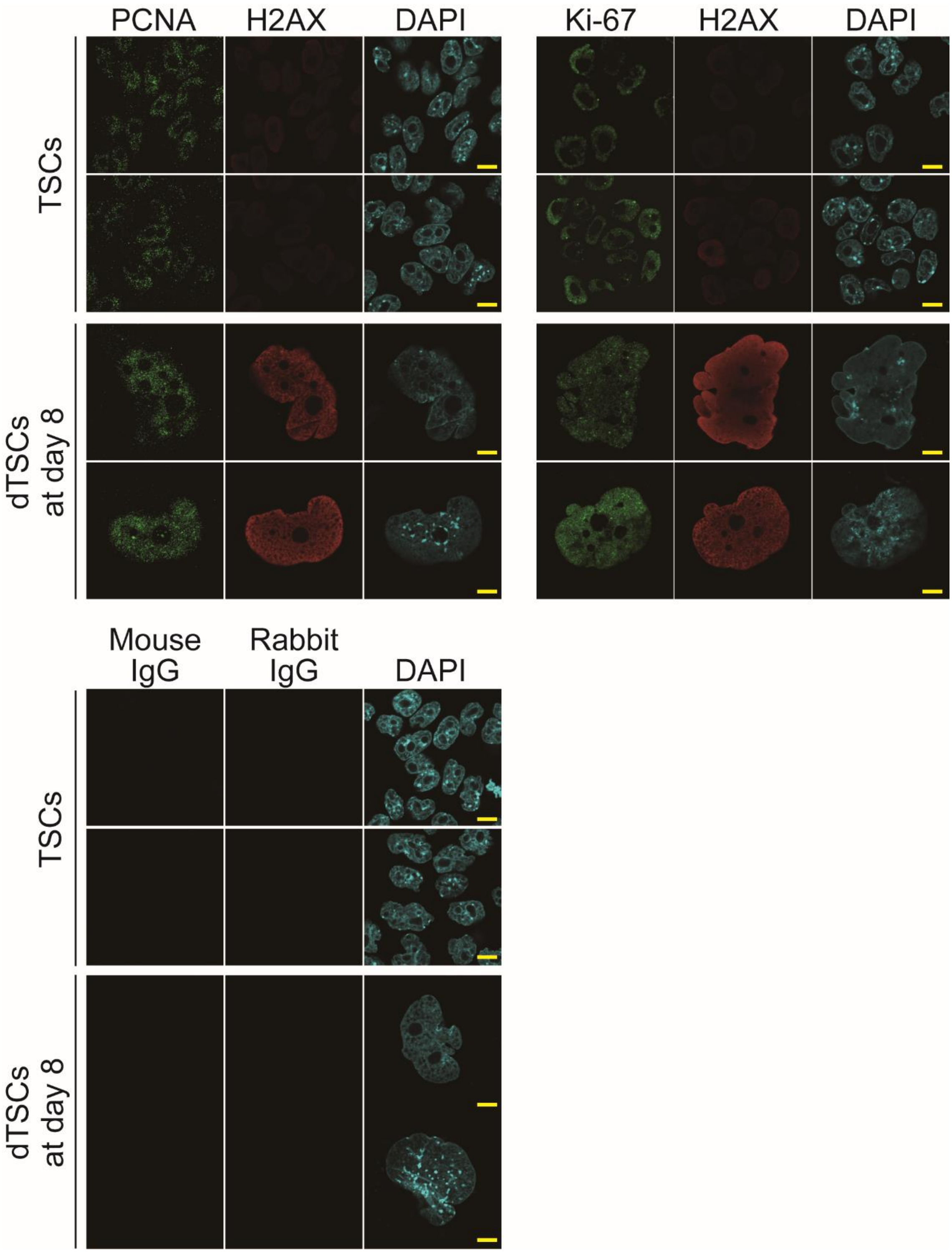

Supplementary figure S4 Expression status of DNA replication markers, PCNA and Ki-67, in TSCs and dTSCs at day 8 of differentiation analyzed by immunofluorescence staining.

Normal mouse IgG and rabbit IgG were used as controls. Bars = 10 μm.

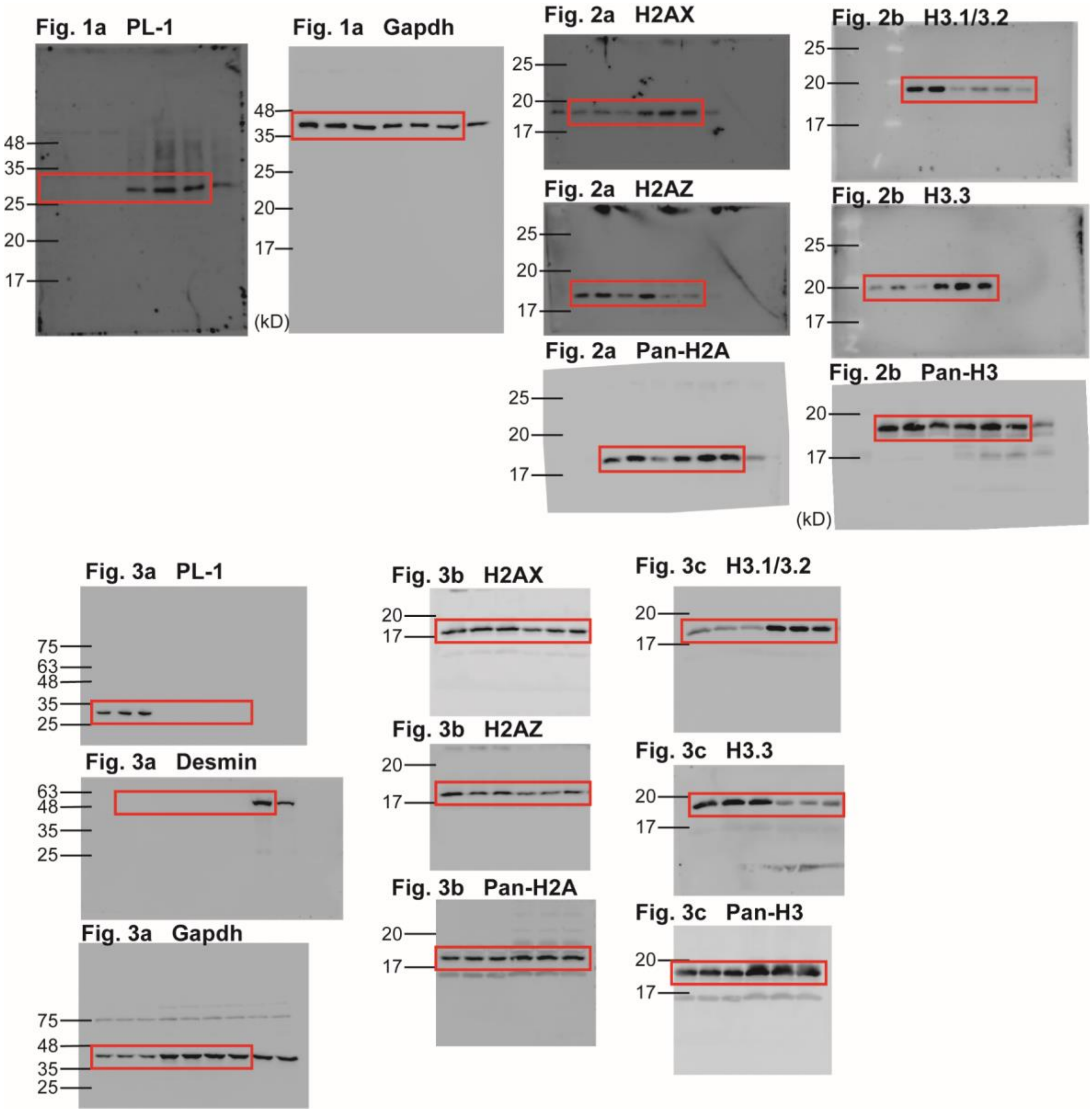

Supplementary figure S5-1 Uncropped blot images

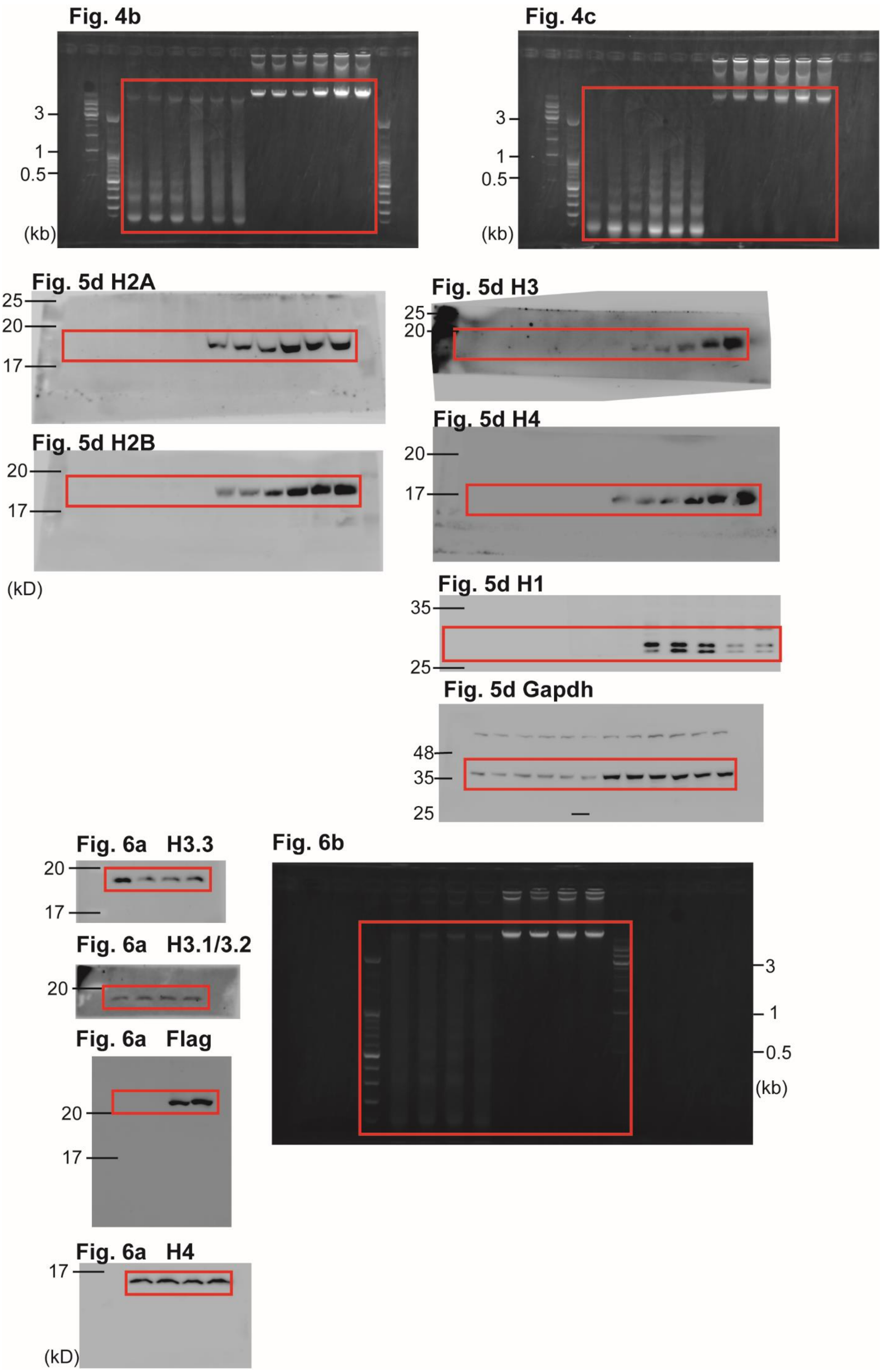

Supplementary figure S5-2 Uncropped blot images

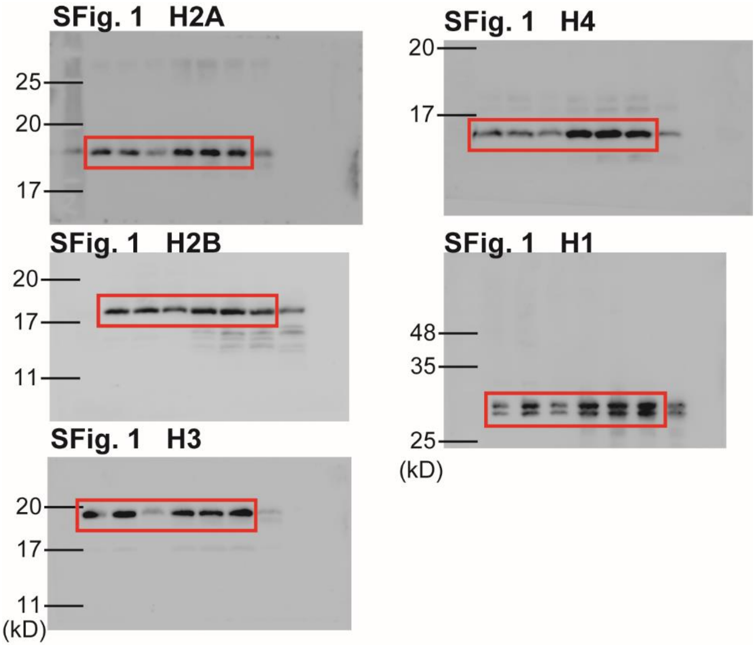

Supplementary figure S5-3 Uncropped blot images

Supplementary movie S1. Dynamics of core histone H4 during TSC differentiation. (**a, b**) H4-GFP was monitored by confocal laser microscope for 96 hours (day 2~6 of differentiation). Representative images from S1A movie was shown in Fig. 4a.
